# Site-specific phosphorylations of the Arf activator GBF1 differentially regulate GBF1 function in Golgi homeostasis and secretion versus cytokinesis

**DOI:** 10.1038/s41598-023-40705-5

**Published:** 2023-08-21

**Authors:** Kendall Walton, Tomasz J. Nawara, Allyson R. Angermeier, Hadley Rosengrant, Eunjoo Lee, Bridge Wynn, Ekaterina Victorova, George Belov, Elizabeth Sztul

**Affiliations:** 1 Department of Cell, Developmental, and Integrative Biology, University of Alabama at Birmingham, 1918 University Boulevard, MCLM 668, Birmingham, AL 35233-2008 USA; 2 Department of Veterinary Medicine, Virginia-Maryland College of Veterinary Medicine, University of Maryland, College Park, MD 20742 USA

**Keywords:** Developmental biology, Cell division, Post-translational modifications, Cell biology, Cell division, Membrane trafficking, Post-translational modifications, Protein transport

## Abstract

Diverse cellular processes, including membrane traffic, lipid homeostasis, cytokinesis, mitochondrial positioning, and cell motility are critically dependent on the Sec7 domain guanine nucleotide exchange factor GBF1. Yet, how the participation of GBF1 in a particular cellular function is regulated is unknown. Here, we show that the phosphorylation of specific highly conserved serine and tyrosine residues within the N-terminal domain of GBF1 differentially regulates its function in maintaining Golgi homeostasis and facilitating secretion versus its role in cytokinesis. Specifically, GBF1 mutants containing single amino acid substitutions that mimic a stably phosphorylated S233, S371, Y377, and Y515 or the S233A mutant that can’t be phosphorylated are fully able to maintain Golgi architecture and support cargo traffic through the secretory pathway when assessed in multiple functional assays. However, the same mutants cause multi-nucleation when expressed in cells, and appear to inhibit the progression through mitosis and the resolution of cytokinetic bridges. Thus, GBF1 participates in distinct interactive networks when mediating Golgi homeostasis and secretion versus facilitating cytokinesis, and GBF1 integration into such networks is differentially regulated by the phosphorylation of specific GBF1 residues.

## Introduction

Cellular homeostasis depends on tightly controlled circuits that engage and coordinate different processes in response to internal and external cues. The integration of cellular metabolic, genetic, and physiological/biochemical state is continuously monitored and relayed to each other for system-wide synchronization. Distinct checkpoints and control mechanisms have evolved to ensure that processes are coordinated in time and space. The secretory pathway, composed of a number of distinct compartments, is tightly regulated to ensure a compensatory anterograde and recycling traffic of membrane and select proteins to ensure continuous transport.

One of the key regulators of the secretory pathway are the small GTPases of the Arf superfamily that facilitate traffic at the ER-Golgi interface^1–5^. Arfs are essential to recruit the heptameric coatomer coat complexes to Golgi membranes to form COPI vesicles that recycle proteins and membrane from the Golgi to the ER^6–9^. All Arfs cycle between GDP- and GTP-bound states and are functional and membrane-associated only when in their GTP-bound (active) state^10^. The GDP/GTP exchange is kinetically unfavorable (to prevent spurious Arf activation) and requires an enzyme, a guanine nucleotide exchange factor (GEF) to catalyze the displacement of the GDP, thus allowing the binding of the activating GTP to the Arf. The GEF responsible for COPI vesicle formation is the Golgi-Specific Brefeldin A Resistance Factor 1 (GBF1), which localizes to the compartments at the ER-Golgi interface, including ER exit sites, ER-Golgi Intermediate Compartment (ERGIC) and the Golgi^11,12^. GBF1 is absolutely essential to activate Arfs required to maintain the architecture and function of the secretory pathway, and the disruption of GBF1 activity with the Brefeldin A (BFA) fungal metabolite or the depletion of cellular GBF1 by RNAi causes Golgi collapse into the ER and inhibits secretion^11,13–16^.

In addition to its well-characterized role in Golgi homeostasis and secretory traffic, GBF1 appears to participate in a multitude of other cellular events spatially removed from the Golgi. GBF1 targets to the leading edge of the plasma membrane in actively moving glioblastoma cells and chemotaxing neutrophils, where its catalytic activity is essential to sustain directional motility in a process involving Rac1-facilitated cortical actin remodeling^17,18^. GBF1 negatively regulates lipid droplet (LD) formation and cellular triacylglycerol content^19^, presumably by facilitating the delivery of ATGL (adipose triglyceride lipase) to LDs through a direct interaction between GBF1 and ATGL^20^. The GBF1-mediated regulation of LD homeostasis appears important to support the successful replication of viruses such as the Dengue Virus^21^. GBF1 also interacts with the mitochondrial membrane protein MIRO to modulate its interactions with the cytoplasmic dynein motors and thereby regulate mitochondrial positioning, as well as impact intra-mitochondrial cristae architecture through an as yet uncharacterized mechanism^22^. The function in mitochondrial positioning is conserved in evolution, as shown by analogous phenotypes caused by GBF1 removal in the worm *C. elegans* and mammalian cells^23^. In addition, GBF1 has been implicated in as yet not understood functions during mitosis^24–27^ in mammalian cells, in support of the septation defects previously described in *S. pombe* deleted of one copy of Gea1, the yeast ortholog of GBF1^28^. In mammalian cells undergoing mitosis, GBF1 is phosphorylated on S292 and S297 by CK2 in late anaphase and early telophase, and so phosphorylated GBF1 appears to localize to the Fleming body of the cytokinetic bridge. The S292/297 phosphorylated GBF1 is degraded during late telophase, but the overall cellular levels of GBF1 do not change, suggesting that only a small fraction of GBF1 is phosphorylated on S292 and S297 and subsequently degraded. Preventing the phosphorylation and the degradation by expressing S292A and S297A causes mitotic defects in cells, with Golgi elements not coalescing correctly in late mitosis. In cells expressing S292A/S297A, the cytokinetic bridge is destabilized, resulting in its collapse and the formation of binucleated cells^26^.

While regulation of Golgi homeostasis and secretory traffic are the main functions of GBF1, the studies describing the importance of GBF1 in other processes, including mitosis suggest that GBF1 performs many distinct functions in a cell. This implies the existence of molecular mechanisms that regulate GBF1 participation in distinct processes. Yet, the determinants that govern GBF1 deployment to a specific process have not been explored.

Herein, we report that phosphorylation of select serine and tyrosine residues within the N-terminal region of GBF1 upstream of the centrally located catalytic Sec7 domain (Sec7d) differentially affects GBF1 ability to support distinct cellular functions. We mined phospho-omics data to identify consistently detected phosphorylation sites within GBF1 and selected only those conserved across GBF1 orthologs for analysis^29^. Using single amino acid substitutions of specific residues, we report that phospho-mimetic substitutions of Serine 233 (S233D), Serine 371 (S371D), Tyrosine 377 (Y377E), and Tyrosine 515 (Y515E) do not affect GBF1 function in maintaining Golgi homeostasis and facilitating secretion, but cause severe defects in cell division. Cell division is also inhibited by the S233A and Y515C substitutions that prevent phosphorylation. The defects occur late during mitosis and affect cytokinesis, with lack of abscission of intercellular bridges leading to the formation of bi- and multi-nucleated cells.

Our results for the first time demonstrate a differential requirement for a particular post-translational modification as a key determinant of GBF1 functionality in a specific cellular process. Our phosphorylation mutants represent unique precision tools that are in stark contrast to the previously used approaches of GBF1 inactivation or removal, which affect all cellular functions of GBF1. Our ability to generate GBF1 mutants compromised in some but not all GBF1 functions forms a critical foundation for future studies to dissect the molecular parameters of GBF1’s role within the secretory pathway away from its activity during cytokinesis.

## Results

### Conserved GBF1 phosphorylation sites

**Figure 1:**
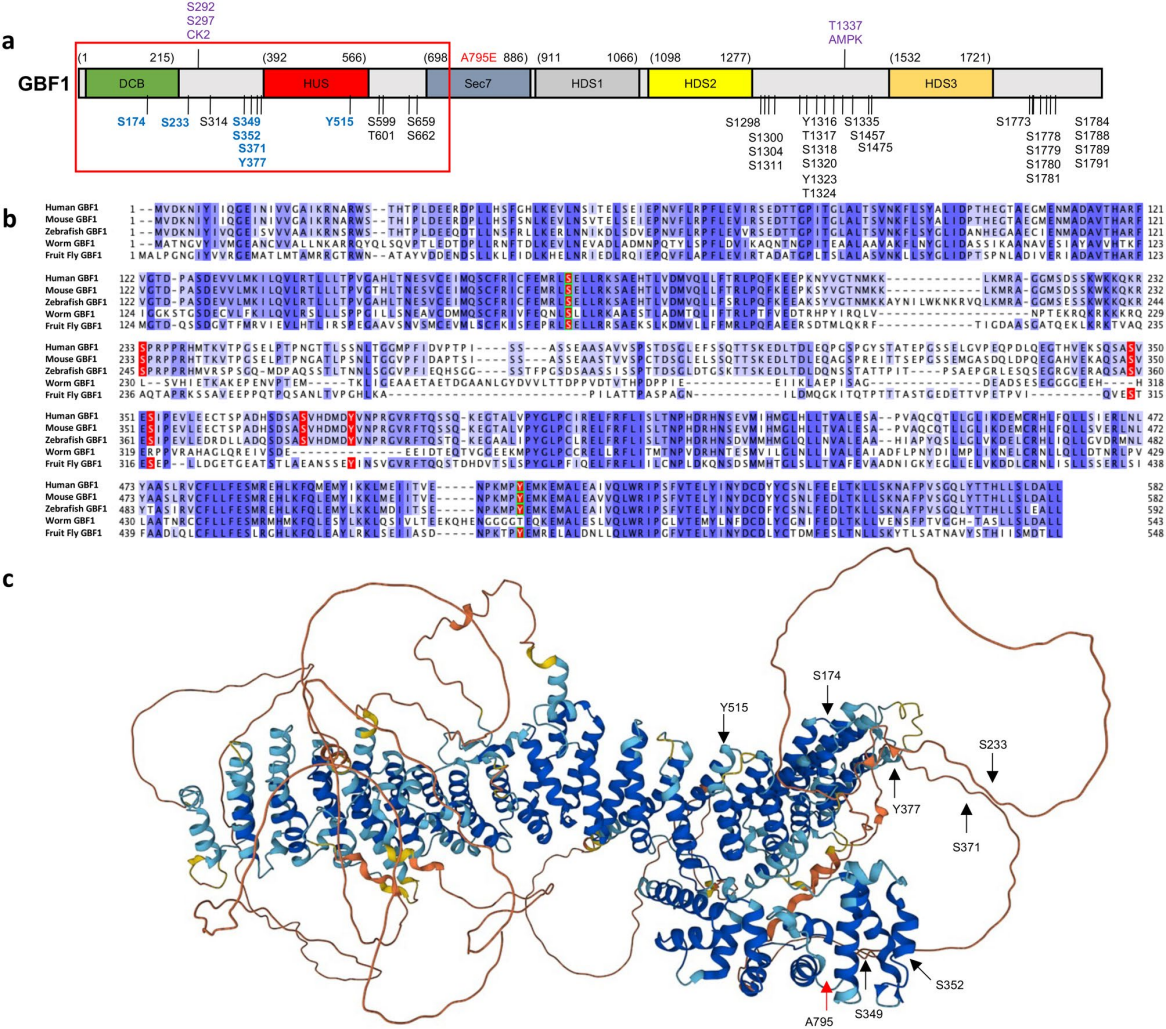
GBF1 phosphorylation sites conserved between species and analyzed in this study. (**a**) Domain architecture of GBF1 with all the reported phosphorylation sites. Highly conserved N-terminal residues chosen for this study are in blue within the red boxed area. All constructs used in this study contain the A795E substitution (in red) to make them BFA resistant. Residues phosphorylated by CK2 or AMPK during mitosis are in purple. (**b**) Alignments of human GBF1 with mouse, zebrafish, worm, and fruit fly GBF1 orthologs showing conserved residues (shaded in blue). The S and Y residues mutated in this study are shaded in red. Residues within the DCB and HUS domains are boxed in green. (**c**) AlphaFold predicted structure of human GBF1 monomer with the phosphorylation sites probed in this study indicated with black arrows. A red arrow points to the A795 residue within the catalytic Sec7 domain that when mutated to E confers BFA resistance.

The stability, localization, and function of proteins is often regulated through reversible phosphorylations of Serine (S), Threonine (T), and Tyrosine (Y) residues that either inhibit protein–protein binding or generate transient binding sites for critical interactions. We have previously queried web-based phospho-omics databases such as PhosphoSitePlus and PhosphoNET and reported on the distribution of GBF1 phosphorylation sites within distinct domains of this multi-domain protein^29^. Herein, we have focused on conserved phosphorylation sites within the N-terminal region of GBF1, upstream of the Sec7d (Fig. 1a, the selected sites are in blue within the boxed region). This subset of phosphorylation sites was selected based on the level of conservation among GBF1 orthologs (Fig. 1b, residues shaded in red; phosphorylated residues 599–662 are not shown because they are not conserved). Some of the selected phosphorylation sites are conserved only across vertebrate species (S233 and S371), while other sites are conserved across species as diverse as human, mice, zebrafish, flies, and worms (S174, S349, S352, Y377, and Y515) (Fig. 1b). The high level of conservation within the GBF1 orthologs suggest that the post-translational phosphorylation of those residues might be relevant to GBF1 function.

Based on the AlphaFold predicted structure of the GBF1 monomer, only two of the 7 phosphorylation sites are within highly structured domains (S174 in DCB and Y515 in HUS) (Fig. 1c, arrows). Both residues are predicted to be surface exposed (S174 is in a loop between α-helix 6 and 7 of DCB and Y515 is within a loop between α-helix 4 and 5 of HUS). The remaining 5 phosphorylation sites are clustered within the relatively poorly conserved linker region between the DCB and HUS domains, suggesting that even the less structured regions of GBF1 may contain regulatory post-translational modifications that could impact selective interactions of GBF1 with its partner proteins.

### Phosphorylation of specific residues modulates GBF1 targeting to the Golgi

To assess the role of phosphorylation in GBF1 function, we generated two mutants for each phosphorylation site—a change to Alanine (A), a residue that can’t be phosphorylated, and a change to a phospho-mimetic, Aspartic acid (D) for phosphorylated Serine (S) and Glutamic acid (E) for phosphorylated Tyrosine (Y)^30^. We are aware that mutating a phosphorylated residue may affect more than just its function as a phospho-acceptor and that the replacement of such residues with negatively-charged amino acids may not mimic the phosphorylated residue in functional terms. Herein we use the terminology of phosphorylation while discussing mutational analysis of a phosphorylated site. For technical reasons, Y515 was mutated to Cysteine (C) instead of A, a residue that also can’t be phosphorylated.

**Figure 2:**
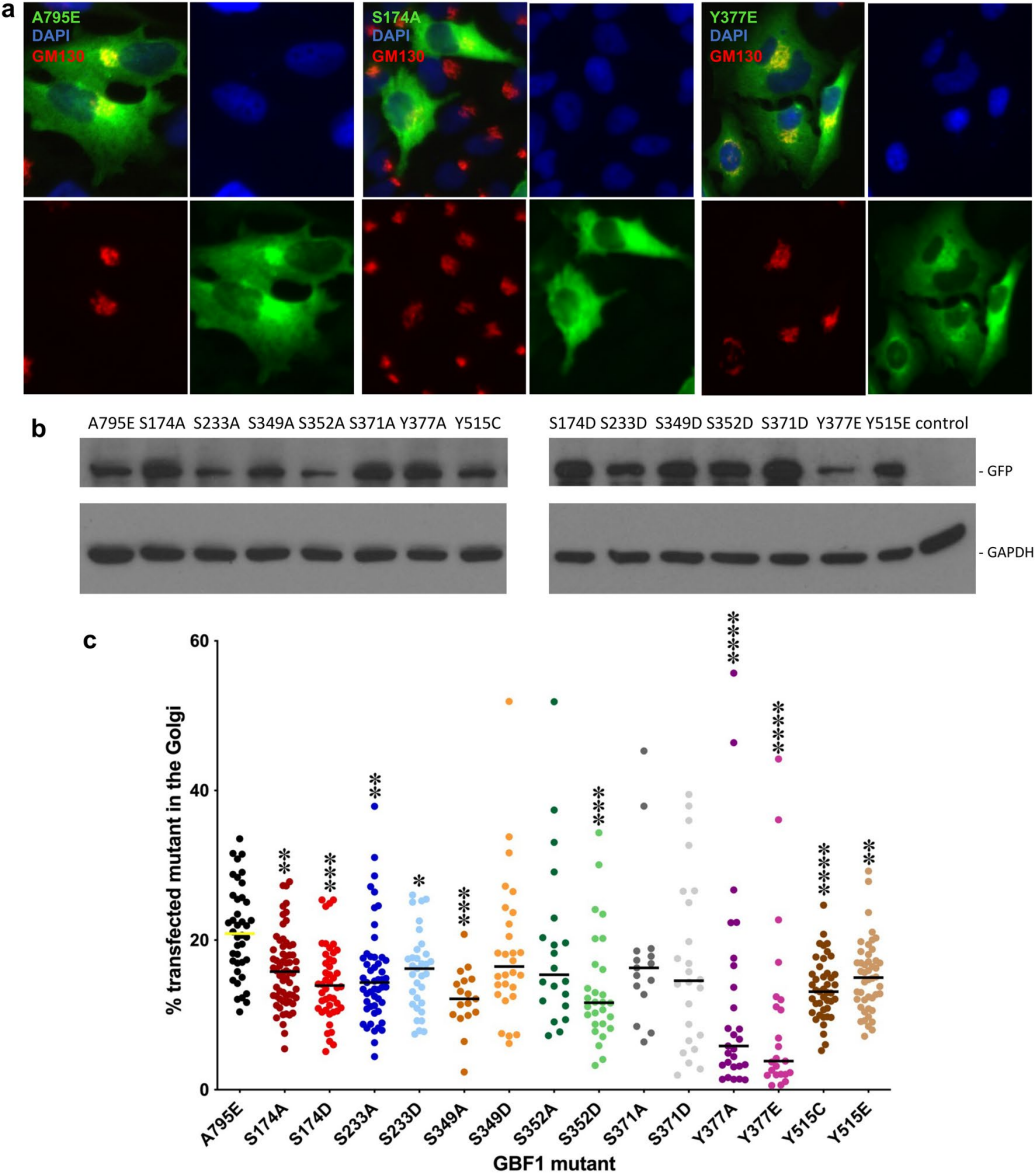
Golgi targeting of GBF1 phosphorylation site mutants. (**a**) HeLa cells were transfected with Venus-tagged GBF1 or a GBF1 phosphorylation site mutant, and after 24 h, the cells were fixed and stained with anti-GFP to detect transfected cells and anti-GM130 to visualize the Golgi (nuclei are stained with DAPI). Representative images of cells transfected with wild-type GBF1 or the indicated GBF1 phosphorylation site mutants show Golgi and cytosolic staining. (**b**) HeLa cells were transfected with Venus-tagged GBF1 or a GBF1 phosphorylation site mutant, and after 24 h, the cells were lysed and the lysates analyzed by SDS-PAGE and immunoblotting with anti-GFP and anti-GAPDH antibodies. A full-length protein of the appropriate molecular weight is expressed in cells transfected with each construct. Blots were cut prior to incubation with antibodies. (**c**) Images analogous to those in (**a**) were used to measure GFP pixel intensity that co-localized with GM130 relative to GFP pixel intensity of the whole cell to obtain the % of total cellular GFP signal that is at the Golgi. The results are from 3 independent transfections and each point represents results from an individual transfected cell. Targeting efficiency of GBF1 phosphorylation mutants relative to that of wild-type GBF1 was compared using the Student’s t-test. N ≥ 15. P < 0.1 (*), P < 0.01 (**), P < 0.001 (***), P < 0.0001 (****).

The expression of each construct after transfection into HeLa cells was assessed by Western blotting with anti-GFP tag antibodies to detect the Venus-tagged construct and anti-GAPDH as a loading control. As shown in Fig. 2b and Supplemental Fig. S1, every construct produced the appropriate molecular weight protein. Because all the constructs are N-terminally tagged and the Western blot was performed with anti-tag antibodies, this indicates that only full-length proteins are present in cells, without fragmentation or partial digestion. The overall expression levels of the majority of the constructs were similar to that of the A795E construct considered wild-type in this analysis, with 3 constructs (S233A, S352A, and Y377E) showing lower overall expression.

The effects of mutating specific phosphorylation sites on GBF1 ability to target to the Golgi was assessed by measuring the amount of exogenously expressed Venus-tagged GBF1 mutant at the Golgi as the % of the expressed protein within the entire cell. Representative images of HeLa cells transfected with Venus-tagged GBF1 or the indicated phosphorylation mutant and stained with anti-GFP to detect transfected cells and the GM130 Golgi marker are shown in Fig. 2a. In this study, wild-type GBF1 (A795E) contains an alanine to glutamate substitution in the catalytic Sec7 domain that confers resistance to Brefeldin A (BFA), but has unmodified phosphorylation sites and is considered wild-type. All of our phosphorylation mutants contain the A795E mutation and all are resistant to BFA.

Golgi targeting analysis was performed for every phosphorylation mutant in this study and the images were used to quantitate the targeting efficiency. Because of the slightly different levels of expression of the different constructs in individual cells, we first assessed the relationship between the level of GBF1 expression and the percent of the cellular GBF1 that associates with the Golgi in cells expressing different levels of GBF1. Five cells (from 3 different images) expressing A795E at low (yellow asterisks in Supplemental Fig. S2), medium (blue asterisks in Supplemental Fig. S2), and high (red asterisks in Supplemental Fig. S2) levels were assessed for the % of total cellular GBF1 present at the Golgi. We found that the % Golgi association appeared linear in cells expressing low to medium levels of GBF1 (Supplemental Fig. S2). In our Golgi targeting analyses, only images of cells with low to moderate levels of expression of each GBF1 construct were used (analogous to cells marked with yellow and blue asterisks in Supplemental Fig. S2). As shown in Fig. 2c, the majority of the phosphorylation mutants exhibit decreased targeting compared to the A795E wild-type GBF1. The most targeting compromised mutants are Y377A, Y377E, and Y515C. It is of particular interest that Y377 and Y515 are both highly conserved in GBF1 orthologs. Conservation often reflects evolutionary pressure to maintain interactive interfaces, suggesting that Y377 and Y515 may form interfaces for GBF1 binding to membrane components.

### Phosphorylation of specific residues decreases GBF1 ability to maintain Golgi architecture

**Figure 3:**
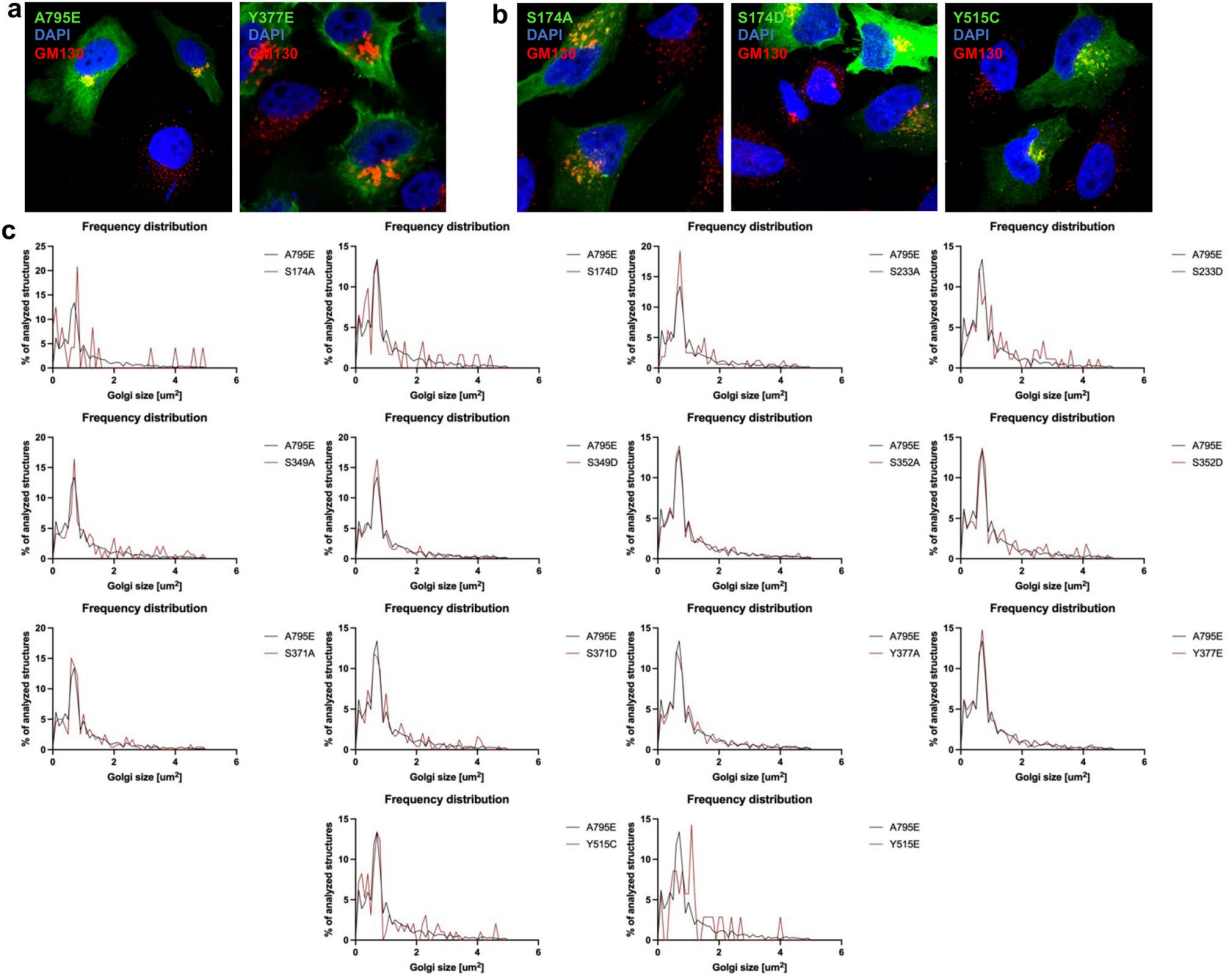
Specific GBF1 phosphorylation site mutants show reduced capacity to support Golgi architecture. HeLa cells were transfected with Venus-tagged wild-type GBF1 or the indicated phosphorylation site mutant. After 24 h, cells were treated with BFA for 1 h to inactivate the endogenous GBF1. Cells were fixed and stained with anti-GFP to detect transfected cells and anti-GM130 to assess Golgi morphology (nuclei are stained with DAPI). (**a**) Representative images of cells expressing Venus-tagged wild-type GBF1 or the Y377E mutant showing the characteristic compact Golgi in the transfected cells. Note the BFA-induced fragmentation of the Golgi in non-transfected cells. (**b**) Representative images of cells expressing Venus-tagged S174A, S174D, or the Y515C mutant showing partially dispersed Golgi elements in the transfected cells. (**c**) Images analogous to those in (**a**) and (**b**) were analyzed and the size distribution of Golgi puncta in cells expressing wild-type GBF1 versus each GBF1 phosphorylation site mutant from 3 separate experiments is represented as frequency distribution.

Golgi structural integrity requires GBF1-dependent Arf activation, as inhibition or depletion of GBF1 leads to Golgi fragmentation^16,31,32^. To assess the ability of each mutant to maintain Golgi morphology, we used a cellular GBF1 “replacement” assay based on the BFA resistance conferred by the A795E mutation within the catalytic Sec7 domain^33^. All the constructs employed in this study contain the A795E mutation and are therefore BFA-resistant. In this assay, untransfected cells show dispersed Golgi due to the inactivation of endogenous GBF1. In contrast, cells expressing wild-type GBF1 containing the A795E mutation maintain compact Golgi, reflecting the functionality of the exogenously expressed GBF1 construct^33^. HeLa cells were transfected with GBF1/A795E or each phosphorylation mutant containing the A795E substitution, and after 24 h treated with BFA for 1 h to inactivate the endogenous GBF1. Cells were then stained with anti-GFP to detect transfected cells and with anti-GM130 to visualize Golgi morphology. As shown in Fig. 3a, cells expressing GBF1 or the Y377E phosphorylation mutant (both containing the A795E substitution) show the characteristic intact Golgi ribbon morphology, indicating that both are functional in supporting Golgi architecture. In contrast, as shown in Fig. 3b, cells expressing the S174A, S174D, or Y515C phosphorylation mutants (all containing the A795E substitution) show a slightly more dispersed pattern of Golgi elements, suggesting that these constructs are functionally compromised.

Analogous images were used to assess the size distribution of Golgi elements in cells expressing each of the phosphorylation mutants and the results are plotted as frequency distributions (Fig. 3c). The majority of the phosphorylation site mutants were able to maintain normal Golgi architecture when present as the only functional GBF1 in the cell, but a higher frequency of smaller Golgi structures was observed in cells expressing the S174A, S174D, and Y515C mutants. This result suggests that these mutants might be partially compromised in their function. The defect could be caused by the decreased Golgi targeting of these mutants (Fig. 2b). In contrast, cells expressing the Y515E mutant showed a shift in frequency distribution towards larger Golgi structures, despite the fact that this construct also exhibited decreased Golgi targeting.

**Figure 4:**
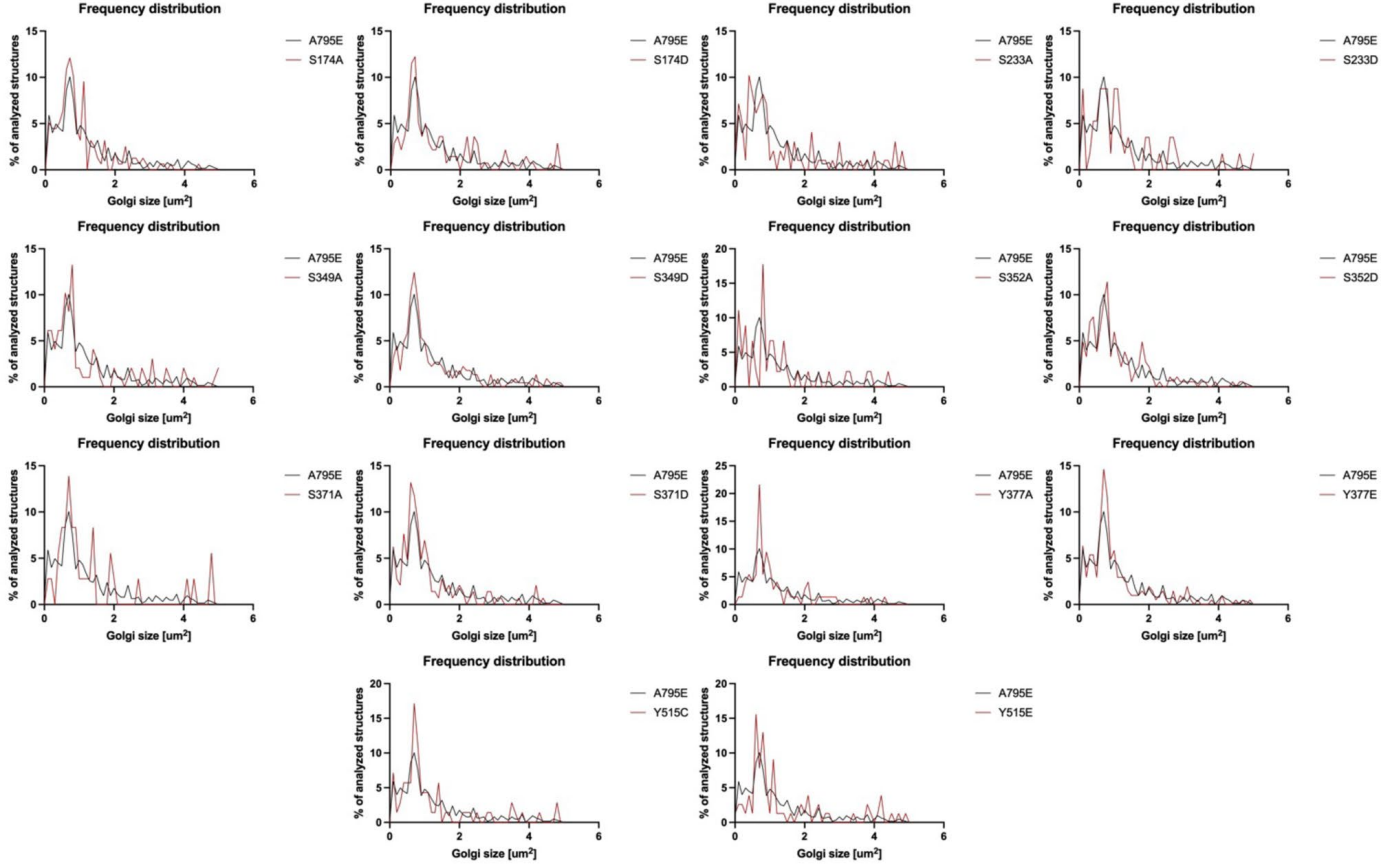
GBF1 phosphorylation site mutants do not act in a dominant negative manner. HeLa cells were transfected with Venus-tagged wild-type GBF1 or the indicated phosphorylation site mutant. After 24 h, cells were fixed and stained with anti-GFP to visualize transfected cells and anti-GM130 to assess Golgi morphology. The size distribution of Golgi puncta in cells expressing wild-type GBF1 versus each phosphorylation mutant from 3 separate experiments is represented as frequency distribution.

We have shown previously that the expression of the catalytically inactive GBF1/E794K^7^ or GBF1/7A^34^ mutants that compete with the endogenous GBF1 for Golgi binding sites disrupts Golgi architecture. To determine whether phosphorylation mutants may impose a dominant negative effect in cells, we assessed whether the mutants might disrupt Golgi architecture in cells containing functional endogenous GBF1. HeLa cells were transfected with Venus-tagged GBF1 or each phosphorylation site mutant and after 24 h stained with anti-GFP to detect transfected cells and with anti-GM130 to visualize Golgi architecture. Golgi intactness was assessed by measuring the number of Golgi elements of a particular size (the size frequency distribution) in cells expressing a particular phosphorylation mutant relative to that in cells expressing the wild-type GBF1 (Fig. 4). Overall, the frequency of Golgi elements of a particular size was similar in cells expressing wild-type GBF1 or the phosphorylation mutants, with the major peaks showing either complete overlap or close proximity. A slight shift towards a higher frequency of smaller Golgi elements was seen in cells expressing the S233A, S233D, S352A, and Y515E mutants. However, even in these cells, the peri-nuclear position of the Golgi was not altered (data not shown), suggesting that the smaller Golgi fragments do engage dynein motors and move in the (-) direction towards the peri-nuclear microtubule-organizing center. Overall, the phosphorylation mutants do not appear to cause significant dominant negative effects on Golgi architecture.

### Expression of specific GBF1 phosphorylation site mutants affects Golgi dynamics but doesn’t affect secretion

**Figure 5:**
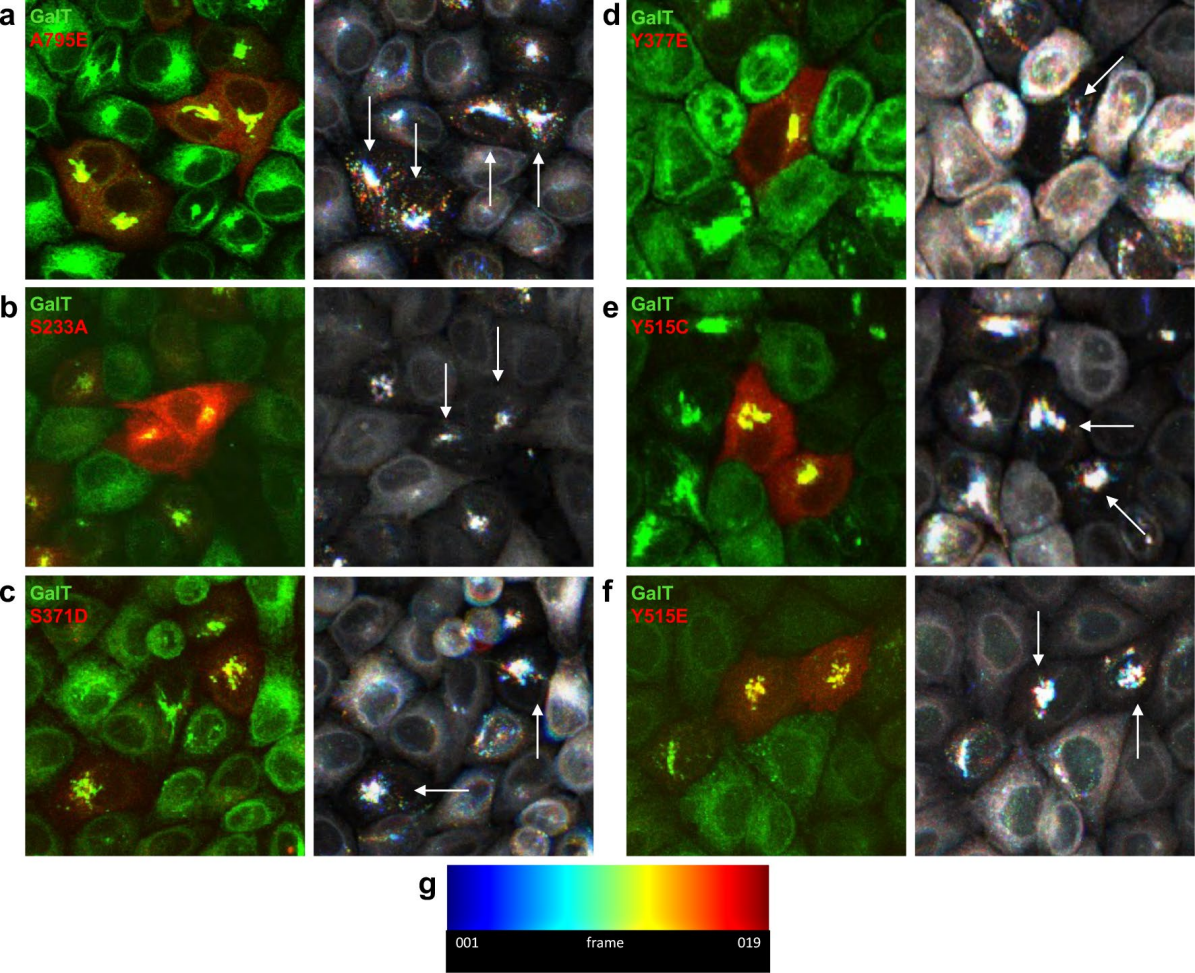
GBF1 phosphorylation site mutants cause changes in Golgi dynamics. HeLa cells stably expressing GFP-tagged GalT were transfected with each Tomato-tagged phosphorylation site mutant for 24 h, treated with BFA for 30 min to inhibit endogenous GBF1 and then live imaged for 1 h in the presence of BFA. The images from each run were combined into a single time course stack, and the dynamics of individual Golgi structures were color coded with white puncta showing minimal movement, while multi color puncta mark structures with different motilities during imaging. (**a**–**g**) Left panels: the localization of each construct at the beginning of live imaging. Right panels: processed image showing movement of Golgi puncta based on the Jet lookup table (**g**) displaying colors associated with movement during subsequent frames. White arrows point to cells expressing wild-type GBF1 or a GBF1 phosphorylation site mutant.

The inability of some GBF1 mutants to efficiently target to the Golgi or to maintain compact Golgi architecture raised the question of whether such mutants may alter Golgi dynamics and/or decrease fusion events needed to coalesce Golgi fragments into a ribbon. To measure the behavior of Golgi structures in cells expressing distinct phosphorylation mutants, HeLa cells stably expressing GFP-tagged Golgi enzyme galactosyl-transferase (GalT) were transfected with each Tomato-tagged phosphorylation construct (all contain the A795E substitution) for 24 h, treated with BFA for 30 min to inhibit endogenous GBF1 and then live imaged for 1 h in the presence of BFA. The spatio-temporal data were combined into a single time course stack, split to extract the GFP (GalT) and the DsRed (phosphorylation mutant) emissions, and the dynamics of individual Golgi structures were color coded. The color reflects each structure’s dynamics with white puncta showing minimal movement, while multi color puncta indicate structures with different motilities during imaging. As shown in Fig. 5a, cells expressing wild-type GBF1 contain a stationary Golgi core (in white) with numerous more distant Golgi puncta (in multiple colors), indicating frequent to and from motility of the elements. In contrast, cells expressing the S233A (Fig. 5b), S371D (Fig. 5c), Y377E (Fig. 5d), Y515C (Fig. 5e), and Y515E (Fig. 5f) phosphorylation mutants predominantly contain white Golgi cores, with less multi-color Golgi puncta, suggesting decreased Golgi fragment dynamics. The lookup table that displays the colors at each individual frame is shown in Fig. 5g. Some of the panels contain cells that appear to be untransfected, but have intact Golgi. However, all cells containing intact Golgi are in fact expressing exogenous GBF1 when the red signal intensity is boosted (data not shown).

**Figure 6:**
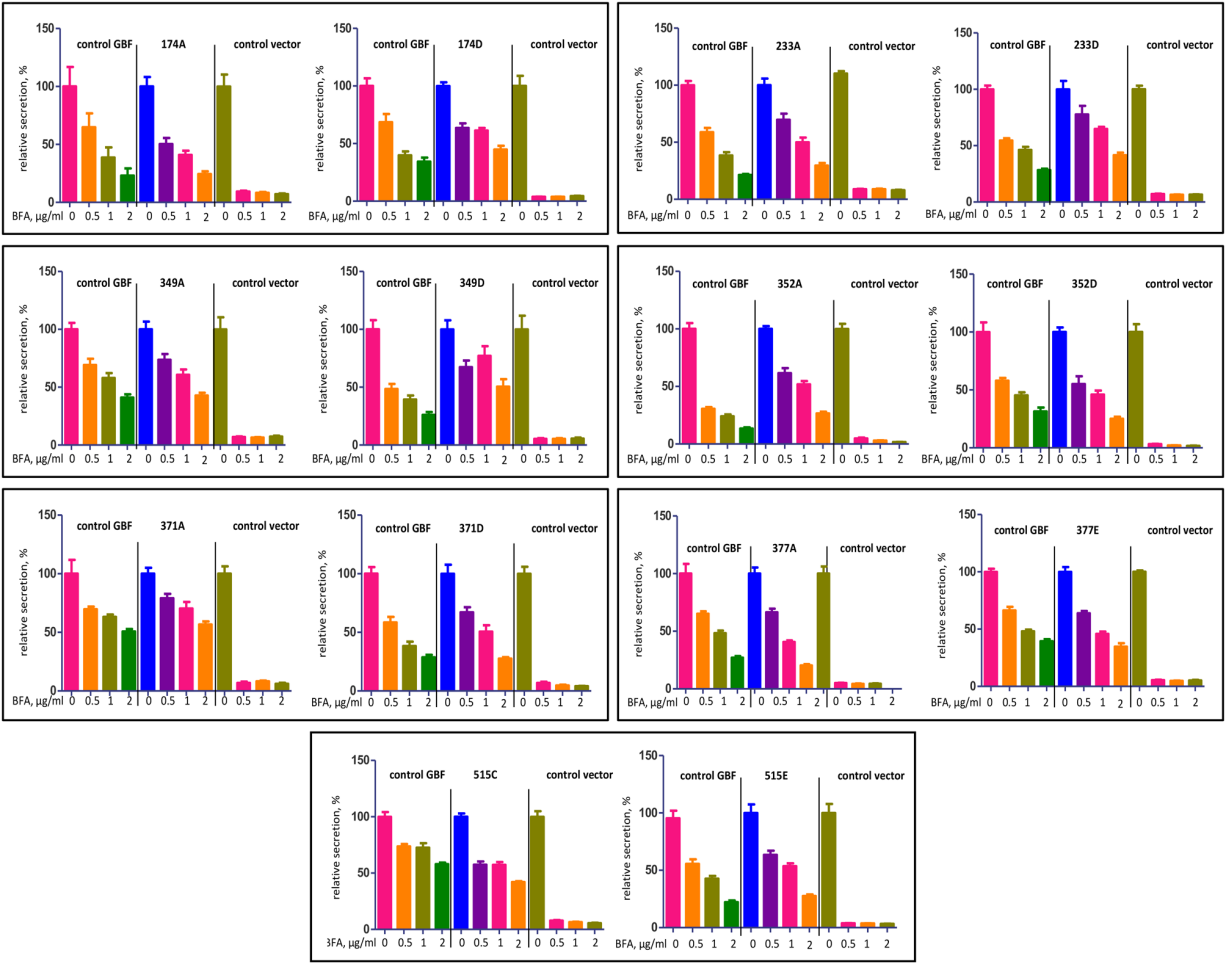
GBF1 phosphorylation site mutants support normal protein secretion. HeLa cells were co-transfected with a Gaussia luciferase reporter and either wild-type GBF1 (control GBF1), the indicated phosphorylation site mutant, or an empty vector (control vector). After 24 h, media was changed to media containing the indicated concentration of BFA (to inactivate endogenous GBF1), and the amount of luciferase released into the media was measured after 4 h. Data for the non-phosphorylatable and a phosphomimetic GBF1 mutant at each phosphorylation site are boxed together.

The reduced capacity of some GBF1 phosphorylation mutants to support Golgi architecture and normal Golgi dynamics prompted us to analyze whether secretion might be compromised in cells expressing these constructs. Secretion of a cargo (a fragment of Gaussia luciferase fused to a leader peptide) was monitored in HeLa cells in a cellular “replacement” assay. In this assay, HeLa cells were co-transfected with the Gaussia plasmid and either A795E (control GBF1), a phosphorylation mutant containing the A795E substitution, or an empty vector (control vector). After 24 h, the cells were put into fresh media containing different concentrations of BFA to inactivate the endogenous GBF1, and secretion of luciferase was monitored over the next 4 h in the presence of BFA to make the exogenously expressed GBF1 mutant the sole functional GBF1 species. As shown in Fig. 6, cells expressing A795E (control GBF1) secrete luciferase even in the presence of the highest BFA concentration, while cells transfected with an empty vector (control vector) are inhibited in secretion even at the lowest concentration of BFA.

Cells expressing each phosphorylation construct (all contain the A795E substitution) exhibited levels of secretion analogous to that of A795E even at the highest concentration of BFA. Normal levels of secretion were observed even for cells expressing phosphorylation mutants that were partially compromised in supporting Golgi homeostasis, in agreement with previous data showing that efficient secretion is compatible with fragmented Golgi architecture, from the highly compacted ribbon structure observed in mammalian cells to the dispersed Golgi mini-stacks present in flies, plants, and yeast^35–37^. Moreover, secretion was fully supported by phosphorylation mutants with reduced Golgi targeting, suggesting that expressing the mutants from a plasmid under the CMV promoter produces sufficient exogenous protein to compensate for their reduced association with Golgi membranes.

### Expression of specific GBF1 phosphorylation mutants causes multinucleation and inhibits cytokinesis

**Figure 7:**
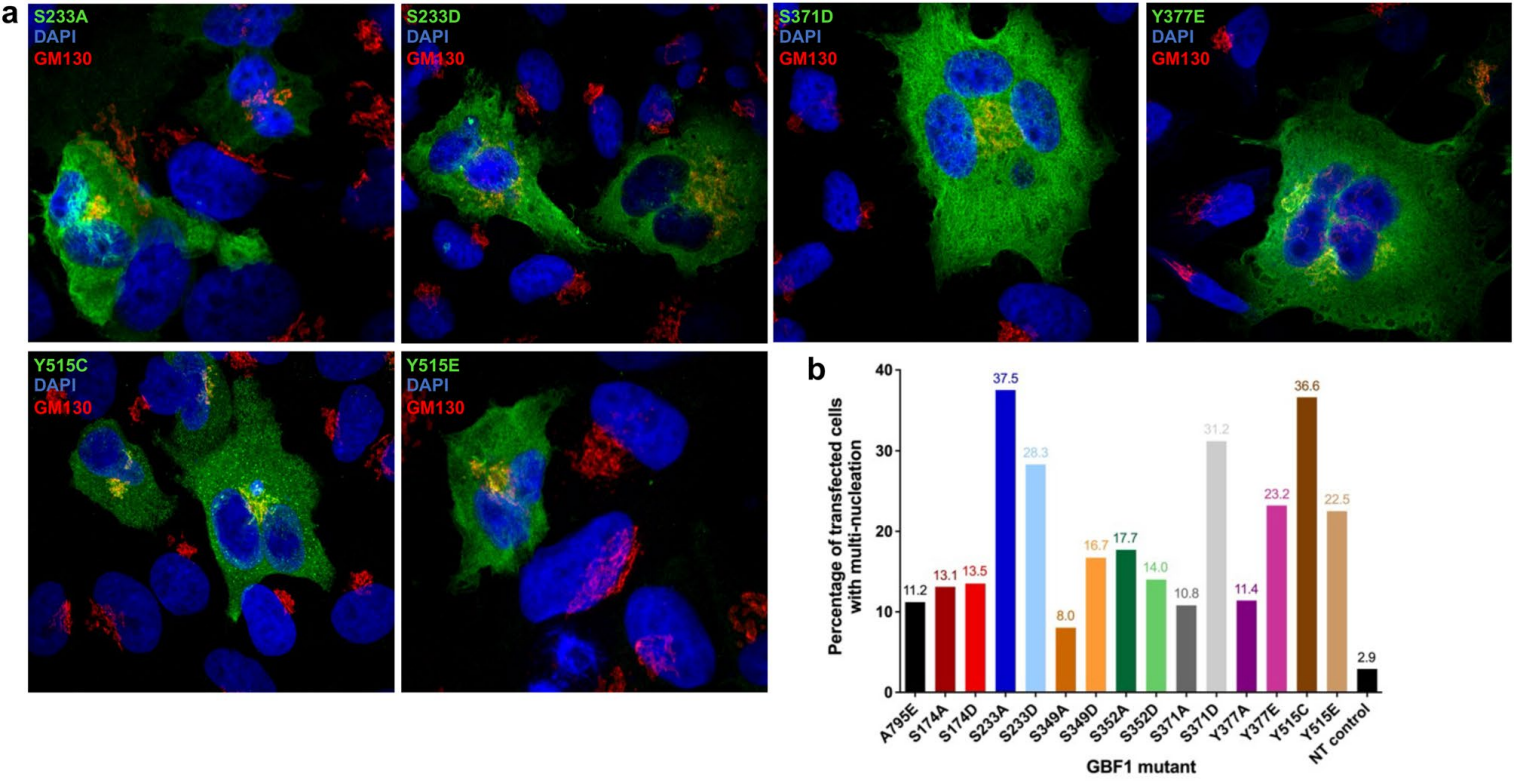
Expression of specific GBF1 phosphorylation site mutants cause multi-nucleation. HeLa cells were transfected with Venus-tagged wild-type GBF1 or the indicated phosphorylation site mutant. After 48 h, cells were stained with anti-GFP to detect transfected cells, anti-GM130 to visualize the Golgi, and DAPI to assess nuclear morphology. (**a**) Representative images of cells expressing phosphorylation site mutants that cause multi-nucleation. Note the single Golgi abutting the two or more nuclei in the transfected cells. (**b**) Quantification of multinucleated cells based on > 50 transfected cells counted in 3 separate experiments. The percentage of transfected cells with two or more nuclei is shown. NT control: % of multi-nucleated non-transfected cells within the same slide.

While examining HeLa cells transfected with a subset of GBF1 phosphorylation mutants, we observed a striking increase in bi- and multi-nucleation relative to cells expressing wild-type GBF1 (Fig. 7a). We quantified the percent of cells containing two or more nuclei to show that while ~ 11.2% of cells transfected with wild-type GBF1 exhibit multi-nucleation, multi-nucleation is dramatically increased (> threefold) in cells expressing specific phosphorylation site mutants (Fig. 7b). In total, 6 phosphorylation site mutants out of the 14 tested in this study increased multi-nucleation. Interestingly, non-transfected cells have a lower rate of multi-nucleation (~ 2.9%), suggesting that even overexpression of wild-type GBF1 increased multi-nucleation.

As shown in Fig. 7b, phosphorylation site mutants with substitutions at S233 or Y515 caused multinucleation irrespective of whether the substituting amino acid couldn’t be phosphorylated (change to A or C) or was a phospho-mimetic (change to D or E). This suggests that GBF1 may participate in critical, but distinct mitotic events when it is dephosphorylated and when it is phosphorylated on S233 and Y515. In contrast, GBF1 with substitutions at S371 or Y377 caused multinucleation only when those amino acids were mutated to a phospho-mimetic, but not when phosphorylation was prevented by an A substitution. This suggests that the continuous phosphorylation of those GBF1 residues interferes with an important mitotic process.

**Figure 8:**
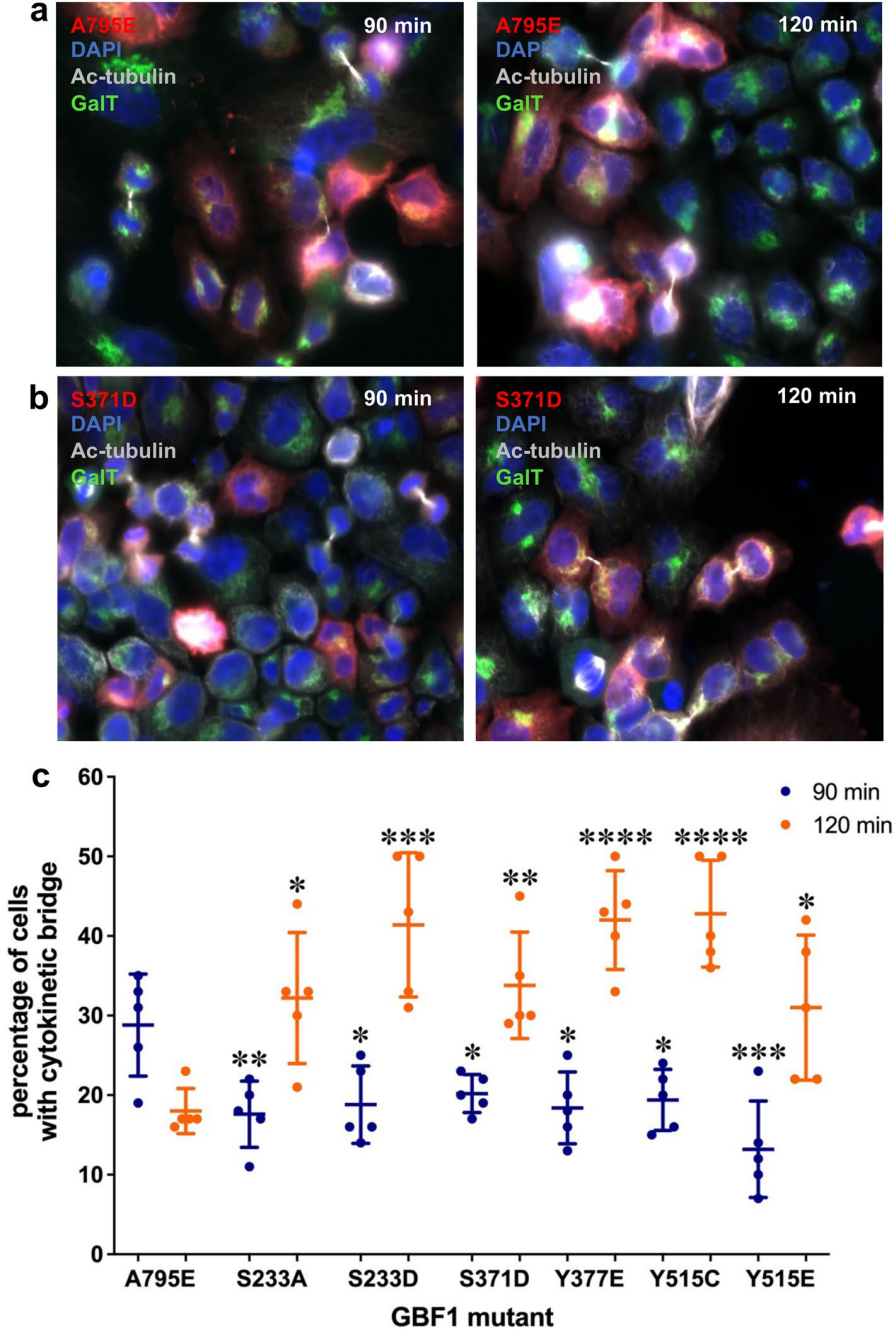
Expression of specific GBF1 phosphorylation site mutants cause a delay in resolution of cytokinetic bridges. HeLa cells stably expressing GFP-tagged GalT were transfected with Tomato-tagged wild-type GBF1 or each multinucleation causing phosphorylation site mutant. After 24 h, cells were treated with RO-3306 for 30 h to arrest at G2/M. The mitotic block was released by a change to fresh media and the cells were fixed 90 or 120 min later. Cells were stained with anti-RFP to detect transfected cells (red), anti-acetylated tubulin to detect cytokinetic bridges (silver), and DAPI to detect the nuclei (blue). (**a**) Representative images of cells expressing wild-type GBF1 after a 90 min and a 120 min release from the G2/M block showing fewer cytokinetic bridges at the later time point. (**b**) Representative images of cells expressing the S371D mutant after a 90 min and a 120 min release from the G2/M block showing the persistence of cytokinetic bridges at the later time point. (**c**) Images analogous to those in (**a**) and (**b**) from 5 random fields (from 3 experiments) for all the phosphorylation site mutants were used to count the number of transfected cells containing cytokinetic bridges. The % of transfected cells that contained a cytokinetic bridge is shown. For statistical analysis, the % of cells expressing a phosphorylation mutant with a cytokinetic bridge at 90 min was compared to the same value for cells expressing wild-type GBF1 at 90 min. Similarly, the % of cells expressing a phosphorylation mutant with a cytokinetic bridge at 120 min was compared to the same value for cells expressing wild-type GBF1 at 120 min. Comparisons were done using a Student’s t-test. P < 0.1 (*), P < 0.01 (**), P < 0.001 (***), P < 0.0001 (****).

To gain insight into the process responsible for the multi-nucleation, we monitored the progression of mitosis in cells transfected with each of the 6 multi-nucleation causing constructs. HeLa cells stably expressing GFP-tagged GalT were transfected with Tomato-tagged wild-type GBF1 or phosphorylation mutant for 24 h. The cells were then synchronized by treatment with RO-3306, a CDK1 inhibitor for 30 h to arrest the cell cycle at G2/M. Subsequently, cells were placed in fresh media to release the mitotic block and allowed to progress through mitosis for 90 or 120 min. Cells were fixed and stained with anti-RFP to identify transfected cells and anti-acetylated tubulin to assess cytokinetic bridge formation. Representative images of cells expressing wild-type GBF1 or the S371D phosphorylation mutant after a 90 and 120 min release from the mitotic block are shown in Fig. 8a,b, respectively.

Analysis of analogous images of cells expressing wild-type GBF1 or phosphorylation site mutants that cause multi-nucleation indicates that ~ 28% of cells expressing wild-type GBF1 contain cytokinetic bridges 90 min after the release of the mitotic block, and this number decreases to ~ 18% at 120 min after release, reflecting the completion of cytokinesis (Fig. 8c). In contrast, 90 min after the release of the mitotic block, cells expressing the multi-nucleation causing mutants contain significantly fewer cytokinetic bridges than cells expressing wild-type GBF1, indicating a mitotic delay. Moreover, at 120 min after the release of the mitotic block, cells expressing phosphorylation mutants have a significantly increased number of cytokinetic bridges, suggesting an additional delay in cytokinetic bridge abscission (Fig. 8c).

**Figure 9:**
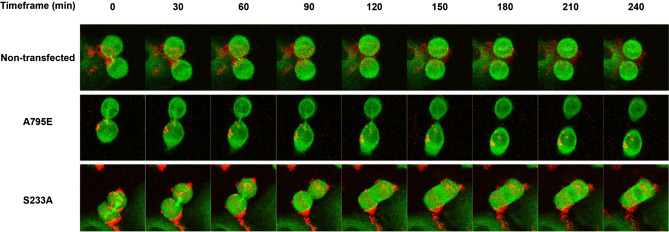
Expression of a GBF1 phosphorylation site mutant causes a disruption in cytokinesis. HeLa cells stably expressing GFP-tagged tubulin were transfected with iRFP-tagged wild-type GBF1 or the S233A phosphorylation site mutant. After 24 h, cells were treated with RO-3306 for 30 h to arrest at G2/M. The mitotic block was released for 60 min, 5 μM biliverdin was added for 30 min and the cells were then imaged for 8 h. Representative frames taken from the time lapse videos show successful cell division of non-transfected control cells and of cells expressing wild-type GBF1. Cells expressing the S233A mutant failed to resolve the cytokinetic bridge and merged together, forming a bi-nucleated cell.

The static immunofluorescence analyses showing a mitotic defect in cells expressing specific GBF1 mutants suggested a defect in cytokinesis. To directly visualize cytokinesis, we analyzed cell division by time lapse microscopy in live cells. HeLa cells stably expressing GFP-tagged tubulin were transfected with infrared fluorescent protein (iRFP)-tagged wild-type GBF1 or the S233A phosphorylation mutant for 24 h. The cells were then synchronized by treatment with RO-3306 for 30 h to arrest at G2/M. Subsequently, cells were placed in fresh media to release the mitotic block and allowed to progress through mitosis for 60 min. Cells were supplemented with 5 μM biliverdin chromophore which acts as a cofactor for iRFP for 30 min, and then imaged for 8 h. We observed successful cytokinetic bridge abscission and cell division in non-transfected cells and in cells transfected with the wild-type GBF1 (Fig. 9). In contrast, cells expressing the S233A mutant failed to break the cytokinetic bridge and collapsed together, resulting in a multi-nucleated cell.

## Discussion

Membrane traffic between distinct subcellular compartments of the secretory and endocytic pathways is a critical function of all eukaryotic cells. Within the secretory pathway, membrane is moved at the ER-Golgi interface by COPII vesicles that traffic in the anterograde direction and by COPI vesicles that recycle key components in the Golgi to ER retrograde direction. The formation of COPI vesicles requires the activation of Arf GTPases through a GDP/GTP exchange process catalyzed by the GBF1 nucleotide exchange factor. The catalytic activity of GBF1 is absolutely essential to maintain COPI vesicle formation, Golgi homeostasis, and secretion.

The activity of many cellular enzymes is regulated through reversible phosphorylations, and herein we assessed whether phosphorylation impacts GBF1 functionality. We have previously described known GBF1 phosphorylation sites^29^, and in this study focused only on sites within the N-terminus of GBF1 and conserved across GBF1 orthologs. Seven sites were mutated either to a non-phosphorylatable residue or to a phospho-mimetic, and the functions of the resulting mutants were compared to that of wild-type GBF1 in a number of cellular assays. We uncovered that most (10 out of 14) of the phosphorylation mutants (both the non- phosphorylatable and the phospho-mimetic) exhibited reduced efficiency of targeting to the Golgi. As the majority of the analyzed phosphorylation sites are within largely unstructured loops within the N-terminus of GBF1, it can be postulated that those regions play a role in the interactions (either intramolecular or with other proteins/lipids) that facilitate Golgi membrane binding.

We assessed the functionality of the GBF1 phosphorylation mutants by a number of criteria. First, we assessed whether the mutants could support Golgi homeostasis when the endogenous GBF1 was inactivated with BFA (all our mutants are engineered to be BFA resistant through the A795E substitution). We uncovered that mutating S174 to either A or D inhibited its ability to support Golgi architecture, leading to fragmented Golgi elements still clustered in the peri-nuclear region (Fig. 3c and Table 1). S174 is within the DCB domain that facilitates GBF1 dimerization, but since dimerization doesn’t appear to be required for GBF1 function^38^, it is possible that mutating S174 impacts GBF1 functionality by a distinct mechanism, perhaps by reducing the ability of the mutant to engage in productive interactions on the membrane. This idea is consistent with the fact that the S174A/D mutants do not act in a dominant negative manner, indicating that they do not efficiently compete with the endogenous GBF1 in cells. A small increase in Golgi fragmentation was also observed in cells expressing the Y515C mutant, and is most likely due to the reduced ability of this mutant to target to the Golgi. All other phosphorylation mutants were able to support Golgi homeostasis when the endogenous GBF1 was inactivated with BFA, suggesting that all these mutants are functional.

**Table 1:** GBF1 functions are differentially regulated by phosphorylation site mutations.

GBF1 phosphorylation mutant	Mutant is unable to maintain Golgi homeostasis in BFA treated cells	Mutant is unable to support secretion	Expression of mutant causes multi-nucleation	Expression of mutant causes reduced dynamics of Golgi fragments
A795E	–	–	–	–
S174A	✓	–	–	*
S174D	✓	–	–	*
S233A	–	–	✓	✓
S233D	–	–	✓	*
S349A	–	–	–	*
S349D	–	–	–	*
S352A	–	–	–	*
S352D	–	–	–	*
S371A	–	–	–	*
S371D	–	–	✓	✓
Y377A	–	–	–	*
Y377E	–	–	✓	✓
Y515C	✓	–	✓	✓
Y515E	✓	–	✓	✓

The inability of the 14 analyzed GBF1 phosphorylation site mutants to support Golgi homeostasis and secretion, cause multi-nucleation, or affect the dynamics of Golgi fragments are indicated by check signs. Only the 5 mutants causing the highest levels of multi-nucleation were assessed for their effects on the dynamics of Golgi elements (asterisks indicate mutants not analyzed for their effect on Golgi dynamics).

Second, we asked whether the mutants could act in a dominant negative manner in the presence of endogenous GBF1, since functionally compromised GBF1 mutants with mutations in the catalytic Sec7 domain (the E794K and the 7A mutants) disrupt Golgi homeostasis and secretion when expressed in cells^7,34^. Minimal disruption in Golgi architecture was seen in cells expressing S233A, S233D, S352A, and Y515E but overall, none of the phosphorylation mutants significantly impacted Golgi homeostasis, suggesting that all the mutants are sufficiently functional to support this activity.

Third, we tested the ability of the GBF1 phosphorylation mutants to maintain an operational secretory pathway. All mutants were fully capable of facilitating the secretion of a soluble fragment of Gaussia luciferase containing a signal peptide that makes it able to enter the secretory pathway^39^ (Table 1). It remains possible that the kinetics of trafficking of specific soluble cargoes or the transport of trans-membrane proteins could be altered in cells expressing the GBF1 phosphorylation mutants and in which endogenous GBF1 is inactivated with BFA. The ability of the mutants to support secretion despite some of them (S174A/D and Y515C) being unable to maintain intact Golgi architecture is consistent with the ability of fragmented Golgi elements to still support secretion^35–37^.

During our analyses, we observed that cells expressing some of the GBF1 phosphorylation mutants were often bi- or multi-nucleated. To probe this phenomenon, we analyzed nucleation in cells expressing the mutants for 48 h, approximately 2 doubling times for HeLa cells^40–42^. The highest induction of multi-nucleation (more than tenfold over that seen in non-transfected control cells) was seen in cells expressing the S233A, S233D, S371D, Y377E, Y515C, and Y515E mutants (Table 1). The multi-nucleated cells contained a single morphologically recognizable Golgi structure abutting the nuclei, consistent with our analyses of normal Golgi morphology and secretion phenotypes in cells expressing these mutants.

A multi-nucleation phenotype often reflects defects during mitosis, and more specifically, complications during cytokinesis. Multi-nucleated cells often can progress through mitosis up to telophase, but are unable to either form or sever the cytokinetic bridge, resulting in the collapse of the daughter cells into a single entity with two duplicated nuclei. We analyzed the formation and resolution of the cytokinetic bridges in cells expressing the 6 multi-nucleation causing GBF1 phosphorylation mutants. The cells were synchronized and blocked at the G2/M stage of mitosis and then released for 90 or 120 min to allow them to recover and progress through the final steps of mitosis. Cells expressing the GBF1 phosphorylation mutants appeared to form cytokinetic bridges later than cells expressing the wild-type GBF1, suggesting that they may proceed through mitosis slower. Moreover, we observed a significant inhibition in the progression of cytokinesis in those cells, with a higher percentage of cells still containing cytokinetic bridges at a time when they were largely resolved in cells expressing wild-type GBF1.

Imaging of cell division by time lapse microscopy confirmed a cytokinetic defect in cells transfected with the S233A multi-nucleation causing mutant. While non-transfected cells and cells expressing wild-type GBF1 formed cytokinetic bridges between the daughter cells and those bridges were subsequently broken allowing the separation of the cells, cells expressing the S233A mutant generated a cytokinetic bridge, but the bridge subsequently collapsed causing the daughter cells to merge into a single, multi-nucleated cell. The small GTPase Rab1 has been previously described as having a role in cytokinesis in Drosophila^43^ by controlling contractile ring constriction. Rab1 has been shown to interact with GBF1 in several studies^44–46^ and perhaps this interaction is disrupted with our GBF1 phosphorylation mutants. Future studies will be needed to address this possibility.

An inhibition in cytokinesis has been previously reported in cells expressing a non-phosphorylatable mutant of GBF1 (S292A/S297A) that stabilizes GBF1 and prevents its degradation^26^. Our comprehensive studies identified additional sites of phosphorylation within GBF1 that when mutated to a phospho-mimetic (the S233D, S371D, Y377E, and Y515E mutants) cause defects in cytokinesis. However, we also document that GBF1 mutants that can’t be phosphorylated (the S233A and Y515C mutants) can cause problems in cytokinesis. We stress that all these mutants inhibit cytokinesis in a dominant negative manner in the presence of functional endogenous GBF1.

A previous study showed that the expression of the non-phosphorylatable S292A/S297A mutant decreased Golgi fragment coalescence during mitosis and arrested cytokinesis^26^. It appears that our mutants that cause multi-nucleation may also affect the dynamics of Golgi elements, and that this may be the cause of the disruption in abscission of the cytokinetic bridge. We tracked the motility of GalT-containing Golgi elements in cells expressing the multi-nucleation causing GBF1 phosphorylation mutants over time and showed an overall reduction in Golgi dynamics in cells expressing the S233A, S371D, Y377E, and either Y515C or Y515E mutants (Table 1). While the molecular basis of the decreased motility remains to be defined, it is possible that the phosphorylation mutants alter the ability of Golgi fragments to couple to microtubule motors, thereby affecting their transport towards the microtubule-organizing center. In mitotic cells, such a motility defect would inhibit the coalescence of Golgi fragments into a single Golgi structure at the centrosome.

The cytokinetic defect observed in our studies is distinct from the well described Golgi disassembly checkpoint, when mitosis is aborted if the Golgi ribbon is not fragmented^47,48^. Normally, the Golgi ribbon disassembles during mitosis in a multi-step process that includes the initial unlinking of the tubular inter-stack continuities during G2, followed by the unstacking of cisterna and the vesiculation of the unlinked cisterna in metaphase. In anaphase, the Golgi reforms, initially re-appearing at two sites as a small Golgi close to the cytokinetic bridge and a larger one at the opposite pole close to the centrosome. In late anaphase, the smaller Golgi migrates towards the centrosome and fuses with the larger Golgi to form a single organelle^49^.

The initial Golgi disassembly is driven by a variety of kinases including MEK1, CDK1, and Plk1 that target Golgi and GRASP proteins to drive Golgi breakdown^50^. Treatments that prevent Golgi fragmentation such as injecting anti-GRASP65 antibodies or expressing non-phosphorylatable mutants of GRASP65 that enhance Golgi stacking in interphase cells prevent the cells from entering mitosis after completing the S phase^51,52^. Similarly, the expression of the constitutively active Arf1 (Q71L) mutant prevents mitotic Golgi disassembly and blocks cytokinesis furrow ingression^53^. Moreover, engineering a physical barrier to Golgi fragmentation by loading the Golgi lumen with 3,3’-diaminobenzidine (DAB) polymers also results in cells arresting at metaphase with circular DNA profiles and monopolar spindles. In these cells, the centrosomes fail to separate, thus preventing normal spindle formation^54^.

The requirement for Golgi disassembly in mitotic progression is also underscored by studies with GBF1 mutants. GBF1 is phosphorylated on Threonine 1337 (T1337) during mitosis by AMPK and this modification is required for GBF1 dissociation from Golgi membranes and the subsequent fragmentation of the Golgi and entry of cells into mitosis^25^. Expression of the non-phosphorylatable T1337A mutant prevents Golgi fragmentation and also prevents entry into mitosis at the G2/M checkpoint, and correlates with the inhibition of chromatin condensation, lack of histone-3 phosphorylation, and a decrease in the number of rounded cells^25^.

Herein, we document a novel function for GBF1 in controlling late stages of mitosis, specifically the events required for cytokinesis, and show that this function is regulated through site-specific phosphorylation. In contrast to the early (pre- or at metaphase) mitotic arrest described previously, our GBF1 phosphorylation mutants do not inhibit Golgi disassembly, but instead inhibit the later cytokinesis step. Our results suggest the existence of a mitotic Golgi reassembly checkpoint that prevents the abscission of the cytokinetic bridge when the cell encounters problems with Golgi reassembly. It also might be relevant that the completion of cytokinesis requires the remodeling of the plasma membrane within the cytokinetic bridge with phosphatidylinositol 4,5-bisphosphate (PIP2) playing an important role^55^. Interestingly GBF1 has been shown to preferentially bind to PIP2^56^, possibly hinting at a transient localization and/or function at the cytokinetic bridge. Future studies will need to explore the molecular mechanisms through which specific GBF1 phosphorylation mutants cause cytokinetic inhibition.

## Materials and methods

### Plasmids

GBF1/A795E was described previously^57^. The pVenus-GBF1 A795E plasmid was a gift from Dr. George Belov (University of Maryland, College Park, MD). The ptdTomato-N1 plasmid was purchased from Takara Bio (Mountain View, CA). The iRFP-713 plasmid was a gift from Dr. Alexa Mattheyses (University of Alabama at Birmingham, Birmingham, AL). GBF1/A795E was ligated into the Tomato plasmid using XhoI and EcoRI restriction enzymes. GBF1/A795E was ligated into the iRFP plasmid using XhoI and SacII restriction enzymes. Point mutations were introduced into the GBF1/A795E Venus, Tomato, and iRFP constructs using the QuikChange XL Site-Directed Mutagenesis Kit purchased from Agilent Technology (Santa Clara, CA). All substitutions were confirmed by sequencing.

### Cell culture and transfection

HeLa cells (ATCC, Manassas, VA), HeLa cells stably transfected with GFP-tagged beta 1,4-galactosyltransferase (GalT) (gift from Dr. Jack Rohrer, Zurich University of Applied Sciences, Wädenswil, Switzerland), and HeLa cells stably transfected with GFP-tagged tubulin (gift from Dr. Ryoma Ohi, University of Michigan, Ann Arbor, MI) were cultured in MEM media (Thermo Scientific, Waltham, MA) supplemented with 10% fetal bovine serum, 1% sodium pyruvate, 1% sodium bicarbonate, 1% PenStrep, and 0.2% NEAA. Cells were grown at 37 °C and 5% CO_2_. Cell transfection was performed with TransIT-LT1 transfection reagent (Mirus Bio, Madison, WI) according to the manufacturer’s instructions.

### Immunofluorescence

In some experiments, cells were incubated with 0.5% BFA (purchased from Cell Signaling Technology, Beverly, MA) for 1 h before processing. Cells were processed for immunofluorescence as follows: cells were washed 3 times in phosphate-buffered saline (PBS), fixed in 3% paraformaldehyde in PBS for 10 min, and quenched with 10 mM ammonium chloride in PBS for 10 min. Cells were permeabilized in 0.1% Triton X-100 in PBS for 7 min. Cells were washed again with PBS. Cells were blocked for 5 min each with 0.4% fish skin gelatin in PBS and 2.5% goat serum in PBS. Cells were incubated with primary antibodies diluted in 0.4% fish skin gelatin for 1 h at room temperature. Cells were washed with PBS containing 0.2% tween-20 (PBST) and blocked as described above. Cells were incubated with secondary antibodies diluted in 2.5% goat serum for 45 min at room temperature. Nuclei were stained with DAPI for 3 min and cells were washed with PBST. Cells were mounted on slides with ProLong Glass antifade mountant (Thermo Scientific, Waltham, MA).

### SDS PAGE and Western blotting

Transfected HeLa cells were lysed in RIPA buffer (150 mM sodium chloride, 1% NP-40, 0.5% sodium deoxycholate, 0.1% SDS, 50 mM Tris pH 8, and 1 mM EDTA) supplemented with a protease inhibitor cocktail. The homogenate was centrifuged at 12,000 rpm for 10 min at 4 °C in a microcentrifuge to remove cell debris. Cell lysates were separated in 8% acrylamide gels and transferred to nitrocellulose membranes. Membranes were blocked with 5% dry milk diluted in 0.1% PBST. Membranes were incubated with primary antibodies diluted in milk/PBST overnight at 4 °C. Membranes were incubated with secondary antibodies diluted in milk/PBST for 45 min at room temperature. Membranes were washed 3 times with PBST and 3 times with PBS. Membranes were incubated with an enhanced chemiluminescent kit for visualization. Rabbit anti-GFP and mouse anti-GAPDH primary antibodies were used at a 1:1000 concentration. Anti-rabbit and mouse HRP-conjugated secondary antibodies were used at a 1:5000 concentration.

### Antibodies

Mouse monoclonal anti-GAPDH (ab8245) and rabbit polyclonal anti-GFP (ab290) were purchased from Abcam (Cambridge, MA). Mouse monoclonal anti-GM130 (610823) was purchased from BD Bioscience (San Jose, CA). Rabbit polyclonal anti-RFP (600-401-379) and rabbit polyclonal anti-GFP (600-401-215) were purchased from Rockland (Philadelphia, PA). Mouse monoclonal anti-acetylated tubulin (T6793) was purchased from Sigma Aldrich (St. Louis, MO). Secondary goat anti-mouse conjugated with Alexa 594 (A32742), Alexa 647 (A21235) and goat anti-rabbit antibodies conjugated with Alexa 488 (A32731) and Alexa 594 (A32740) were purchased from Thermo Scientific (Waltham, MA). Secondary goat anti-mouse and goat anti-rabbit antibodies conjugated to HRP were purchased from SouthernBiotech (Birmingham, AL).

### Protein secretion assay

HeLa cells were cotransfected in 96-well plates with plasmids encoding Gaussia luciferase (GLuc) and the GBF1 mutants. An empty vector was used as a negative control. The next day, the medium was removed, and the cells were washed to remove secreted luciferase and incubated in 25 μl of fresh medium supplemented with the indicated amount of BFA. After 4 h of incubation, the medium was transferred into another 96-well plate, and the amount of secreted luciferase was measured with a BioLux Gaussia Luciferase Assay Kit (New England BioLabs, Ipswich, MA) according to the manufacturer’s recommendations.

### Cell synchronization

HeLa cells were treated with 10 μM of the CDK1 inhibitor RO-3306 (Sigma Aldrich, St. Louis, MO) to sync cells in G2. Cells were placed in an incubator for 30 h at 37 °C and 5% CO_2_. Cells were washed 3 times with prewarmed PBS to release the cells from the G2 block. Cells were incubated with fresh culture media for different time points to allow the cell cycle to proceed.

### Microscopy and live imaging

Cell images were taken with a Leitz-Wetzlar Type 307 microscope equipped with a 12-bit CCD camera and a Nikon Eclipse Ti2 microscope. Fluorescence channels were merged using ImageJ software. Timelapse imaging was conducted with HeLa cells stably expressing GFP-tagged GalT and HeLa cells stably expressing GFP-tubulin on an Olympus FluoView1000 using a 20x/0.95 NA water objective. Z-stack slices were optimized for the objective at 1.18 mm thickness. For the duration of the imaging, cells were on a 37 °C heat plate and maintained in L-15 media (Thermo Scientific, Waltham, MA) supplemented with 10% fetal bovine serum. Cells transfected with iRFP-tagged plasmids were incubated with 5 μM biliverdin (Cayman Chemical, Ann Arbor, MI) for 30 min prior to imaging.

### Assessing the relationship between expression levels and Golgi targeting

Immunofluorescence images taken as described above were opened using ImageJ. Channels were split using the Split Channels function. Golgi was located in the red GM130 channel and the GFP pixel intensity was measured in this location in the green GFP channel using the freehand selection and measure functions. The GFP pixel intensity of the entire cell was measured using the same freehand selection and measure functions. The ratio of GFP pixel intensity at the Golgi was then calculated.

### Size frequency distribution

Immunofluorescence images were opened using MATLAB. Background fluorescence was eliminated by running the images with an Otsu algorithm for thresholding. Green fluorescence signal that indicates transfected cells was extracted using a combination of masking filters for the green channel. Red fluorescence signal that indicates Golgi particles was extracted using a combination of masking filters for the red channel. The channels were combined and red pixels contained inside the green channel were measured and plotted on a distribution graph by size and frequency.

### Golgi dynamics analysis

Live cell imaging movie files of the HeLa GalT cells were open with Fiji software and combined to a single time course stack and split to extract the GFP emission. The background was measured and bleach correction was performed using the simple ratio method. The image stack was then registered, to correct for drift during imaging, using the MultiStackReg and the Rigid Body approach. The temporal-color code plugin was used with a Jet lookup table to label the Golgi fragments based on movement.

### Statistical analysis

The percentages of GBF1 Golgi targeting, multi-nucleated cells, and cells with cytokinetic bridges were calculated using Prism software. Data sets were compared using the Student’s t-test.

### Supplementary Information

Supplementary Figure 1.

Supplementary Figure 2.

Supplementary Legends.

Supplementary Video 1.

Supplementary Video 2.

Supplementary Video 3.

## Data Availability

All data obtained in this study will be available from the corresponding author by written request.
