# Supplementary material for: Site-specific phosphorylations of the Arf activator GBF1 differentially regulate GBF1 function in Golgi homeostasis and secretion versus cytokinesis
Source: Sci Rep. 2023 Aug 21;13:13609. doi: 10.1038/s41598-023-40705-5 (PMC10442430; doi:10.1038/s41598-023-40705-5)
Supplement: Supplementary file 3 — Supplementary Legends. [file 41598_2023_40705_MOESM3_ESM.docx]

**Supplemental Figure 1. Expression of GBF1 P site mutants**

HeLa cells were transfected with Venus-tagged GBF1 or a GBF1 phosphorylation site mutant, and after 24 hours, the cells were lysed and the lysates analyzed by SDS-PAGE and immunoblotting with anti-GFP and anti-GAPDH antibodies. A full-length protein of the appropriate molecular weight is expressed in cells transfected with each construct. Blots were cut prior to incubation with antibodies.

**Supplemental Figure 2. Linear increase in Golgi targeting of GBF1 mutants in cells expressing GBF1 at different levels.**

HeLa cells were transfected with Venus-tagged wild-type GBF1, and after 24 hours, the cells were fixed and stained with anti-GFP to detect transfected cells and anti-GM130 to visualize the Golgi (nuclei are stained with DAPI). 5 cells with different levels of GBF1 expression (low, medium, and high) were assessed for Golgi targeting of GBF1. Golgi targeting was calculated as a percentage of total cellular GFP signal detected at the Golgi. Low expressing cells are marked with yellow asterisks. Medium expressing cells are marked with blue asterisks. High expressing cells are marked with red asterisks. Averages from n=5 were calculated for each category and are listed.

**Supplemental Movie 1. Normal cytokinesis in non-transfected cells.**

HeLa cells stably expressing GFP-tagged tubulin were mock transfected. After 24 hours, cells were treated with RO-3306 for 30 hours to arrest at G2/M. The mitotic block was released for 60 minutes, 5 μM biliverdin was added for 30 minutes, and the cells were then imaged for 8 hours. The time lapse video shows successful cell division of non-transfected control cells.

**Supplemental Movie 2. Normal cytokinesis in cells expressing wild-type GBF1.**

HeLa cells stably expressing GFP-tagged tubulin were transfected with iRFP-tagged wild-type GBF1. After 24 hours, cells were treated with RO-3306 for 30 hours to arrest at G2/M. The mitotic block was released for 60 minutes, 5 μM biliverdin was added for 30 minutes, and the cells were then imaged for 8 hours. The time lapse video shows successful cell division of cells expressing wild-type GBF1.

**Supplemental Movie 3. Expression of a GBF1 phosphorylation site mutant disrupts cytokinesis.**

HeLa cells stably expressing GFP-tagged tubulin were transfected with iRFP-tagged S233A phosphorylation site mutant. After 24 hours, cells were treated with RO-3306 for 30 hours to arrest at G2/M. The mitotic block was released for 60 minutes, 5 μM biliverdin was added for 30 minutes, and the cells were then imaged for 8 hours. The time lapse video shows disruption of cytokinesis of cells expressing the S233A mutant, with a failure in the abscission of the cytokinetic bridge and the collapse of the daughter cells to form a single bi-nucleated cell.
